# Hydroxychloroquine Use and the Risk of Breast Cancer in Women With Systemic Lupus Erythematosus: A Systematic Review With No Eligible Studies

**DOI:** 10.7759/cureus.101627

**Published:** 2026-01-15

**Authors:** Ryuichi Ohta, Taichi Fujimori, Kunihiro Ichinose

**Affiliations:** 1 Community Care, Unnan City Hospital, Unnan, JPN; 2 Rheumatology, Shimane University Faculty of Medicine, Izumo, JPN

**Keywords:** breast cancer, cancer risk, evidence gap, hydroxychloroquine, pharmacoepidemiology, systematic review, systemic lupus erythematosus

## Abstract

Hydroxychloroquine (HCQ) is a cornerstone therapy for systemic lupus erythematosus (SLE) and is frequently prescribed for long-term use, particularly in women. Although patients with SLE have been reported to have altered risks of malignancy, including breast cancer, the potential association between HCQ exposure and breast cancer incidence has not been clearly established. Given the widespread and prolonged use of HCQ, clarification of its long-term safety profile for cancer is clinically important.

We conducted a systematic review in accordance with the Preferred Reporting Items for Systematic Reviews and Meta-Analyses (PRISMA) 2020 guidelines to evaluate the association between HCQ use and breast cancer risk in women with SLE. PubMed (MEDLINE), Embase, and Web of Science were searched from inception to December 29, 2025, without language restrictions. Eligible studies were required to include women with SLE, evaluate HCQ use as an independent exposure, report breast cancer incidence as an outcome, and provide extractable effect estimates. Two reviewers independently screened titles, abstracts, and full texts. The search identified 548 records; after removing 73 duplicates, 475 were screened, and 469 were excluded. Six full-text articles were assessed in detail, but none met the predefined inclusion criteria, most commonly due to a lack of independent assessment of HCQ exposure or the absence of breast cancer-specific outcome analyses. Consequently, no studies were included in the final review.

Despite extensive epidemiological research on cancer risk in SLE, no studies have directly evaluated the association between HCQ use and breast cancer risk in women with SLE. From an epidemiological standpoint, the absence of eligible studies is not unexpected, given the relatively low incidence of breast cancer in SLE populations and the widespread use of HCQ as standard-of-care therapy.

A broader analytic strategy - such as evaluating overall malignancy risk associated with HCQ exposure with site-specific cancers examined as secondary outcomes - may represent a more efficient approach for future studies. Nevertheless, the lack of breast cancer-specific analyses treating HCQ as an independent exposure highlights an important gap in the literature, particularly given the frequency with which this question arises in clinical counseling of women with SLE.

## Introduction and background

Systemic lupus erythematosus (SLE) is a chronic autoimmune disease predominantly affecting women and characterized by systemic inflammation, immune dysregulation, and long-term exposure to immunomodulatory therapies [1]. Advances in treatment have substantially improved survival, resulting in an aging SLE population in which long-term comorbidities, including malignancy, have become increasingly relevant clinical concerns [2].

Previous epidemiological studies have consistently demonstrated that patients with SLE have an elevated overall risk of malignancy compared with the general population, particularly for hematological cancers such as non-Hodgkin lymphoma, whereas the incidence of breast cancer - a hormonally regulated malignancy predominantly affecting women - has been reported as unchanged or reduced in this population [3-7]. In contrast, hormone-sensitive cancers, including breast cancer, have been reported to occur at similar or even lower rates in women with SLE than in the general population [6]. Large population-based cohort studies from Nordic countries and meta-analyses have suggested a reduced or unchanged standardized incidence ratio for breast cancer among women with SLE, although the underlying mechanisms remain incompletely understood [7]. This paradoxical epidemiological pattern - occurring in a population with chronic immune dysregulation and prolonged exposure to immunomodulatory therapy - raises clinically relevant questions regarding the role of long-term treatments such as hydroxychloroquine (HCQ) in modifying breast cancer risk, particularly in women.

HCQ is a cornerstone therapy for the management of SLE and is recommended for long-term use in most patients due to its disease-modifying effects, reduced disease flares, and favorable safety profile [8]. Beyond its immunomodulatory properties, HCQ has attracted attention for its potential antineoplastic effects, including inhibition of autophagy, modulation of tumor microenvironments, and enhancement of antitumor immune responses in preclinical and oncological studies [9,10]. These biological mechanisms have raised clinically relevant questions regarding whether long-term HCQ exposure may influence cancer development, including breast cancer, in patients with autoimmune diseases.

Despite extensive literature examining cancer risk in SLE, most epidemiological studies have focused on the association between SLE itself and malignancy or on the potential carcinogenic effects of cytotoxic immunosuppressive agents such as cyclophosphamide and azathioprine [11,12]. In these studies, HCQ has typically been treated as background therapy or as a covariate rather than as an independent exposure of interest. Consequently, primary epidemiological studies have not directly evaluated the association between HCQ use and site-specific cancer incidence - particularly breast cancer - in women with SLE.

Given the widespread and prolonged use of HCQ in women with SLE, clarifying its potential relationship with breast cancer risk is of substantial clinical importance for patient counseling, long-term safety evaluation, and shared decision-making. Breast cancer was selected as the outcome of interest because it is a hormonally regulated malignancy predominantly affecting women, remains a major public health concern, and has shown a paradoxically reduced or unchanged incidence in SLE populations despite chronic immune dysregulation and long-term immunomodulatory therapy. To our knowledge, no systematic review has specifically examined whether HCQ exposure is associated with breast cancer incidence in women with SLE.

Therefore, we conducted a systematic review to evaluate the existing epidemiological evidence on the association between HCQ use and breast cancer risk in women with SLE, and to identify gaps in the current literature that warrant future investigation.

## Review

Methods

Study Design

This review was conducted in accordance with the methodological framework outlined in the Preferred Reporting Items for Systematic Reviews and Meta-Analyses (PRISMA) 2020 guideline [13]. It aimed to systematically identify and evaluate epidemiological studies examining the association between HCQ exposure and breast cancer incidence among women with SLE. Because substantial heterogeneity in study design and outcome reporting was anticipated, quantitative synthesis was not planned.

Protocol Registration

The review protocol was registered in the International Prospective Register of Systematic Reviews (PROSPERO). The registration number is CRD420251270848.

Search Strategy

A systematic literature search was performed across PubMed (MEDLINE), Embase, and Web of Science from database inception through November 2025, with no language restrictions. The search strategy combined controlled vocabulary terms and free-text keywords relating to systemic lupus erythematosus, hydroxychloroquine, antimalarial agents, and breast cancer. In PubMed, Medical Subject Headings (MeSH) terms, including Systemic Lupus Erythematosus and Antimalarials, were used alongside free-text terms. In Embase, corresponding Emtree terms, including “systemic lupus erythematosus” and “antimalarial agent’”exp, were incorporated. In Web of Science, disease-related search terms included “systemic lupus erythematosus,” “SLE,” and “lupus.” Across all databases, cancer-related terms included “breast cancer,” “breast neoplasm,” and “malignancy.” Reference lists of relevant review articles and eligible full-text publications were also manually screened. Detailed, reproducible search strategies for each database are provided in Appendix 1.

Study Selection

Studies were eligible for inclusion if they met all prespecified criteria. Eligible studies were required to involve women diagnosed with SLE and to assess HCQ exposure as a variable of interest, either as the primary exposure or as an independently analyzed factor. In addition, studies had to report breast cancer incidence as an outcome and provide extractable effect estimates - such as hazard ratios, risk ratios, or odds ratios - or sufficient data to derive these measures. Only observational study designs, including cohort, case-control, and nested case-control studies, were considered.

Articles were excluded if they were reviews, meta-analyses, case reports, conference abstracts, animal studies, or if they did not specifically evaluate HCQ exposure or breast cancer-related outcomes. Title and abstract screening were performed independently by two reviewers, followed by full-text assessment of potentially relevant articles. Disagreements were resolved through discussion until consensus was achieved.

Data Extraction and Synthesis

A standardized data extraction framework was prepared in advance to capture key study characteristics, including study design, participant demographics, definitions of HCQ exposure, assessment of breast cancer outcomes, duration of follow-up, and reported effect estimates. As no studies fulfilled the predefined eligibility criteria, data extraction and qualitative synthesis were ultimately not undertaken. Nevertheless, reasons for exclusion at the full-text review stage were systematically documented and categorized to enhance the transparency and reproducibility of the study selection process.

Statistical Analysis

Because no studies met the predefined inclusion criteria, a meta-analysis was not performed. As a result, quantitative synthesis and formal assessment of statistical heterogeneity were not feasible. The findings are therefore presented descriptively, emphasizing the absence of eligible studies and summarizing the nature of the excluded literature.

Results

Study Selection

**Figure 1:**
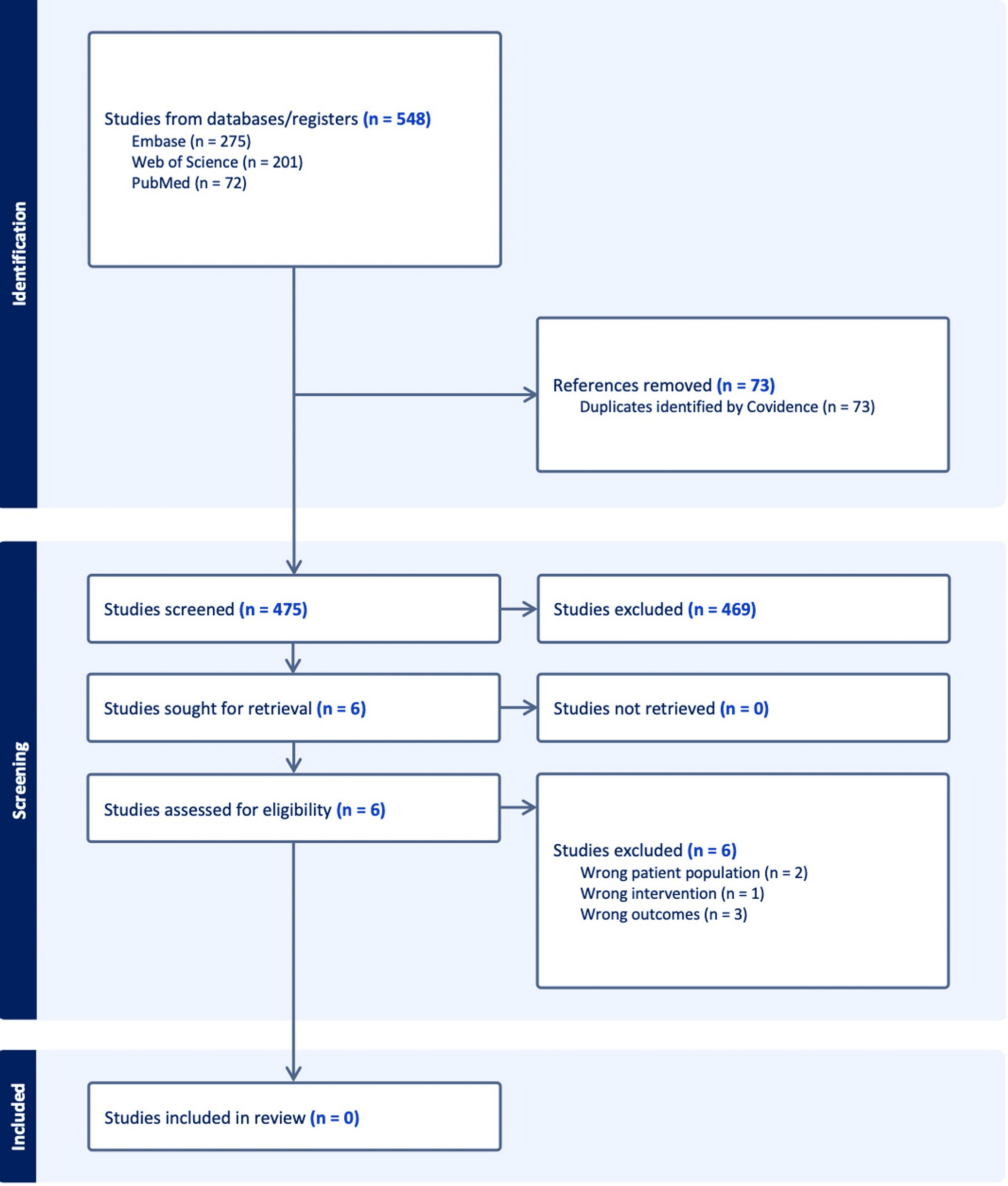
Selection Flow The figure presents the PRISMA 2020 flow diagram illustrating the study selection process. The figure details the identification of records from electronic databases, removal of duplicates, title and abstract screening, and the final determination that no eligible studies met the predefined criteria for inclusion.

The study selection process is illustrated in Figure 1. The database search yielded a total of 548 records, comprising 275 from Embase, 201 from Web of Science, and 72 from PubMed. After the removal of 73 duplicate records using Covidence, 475 unique records remained and were screened by title and abstract. Of these, 469 records were excluded for not meeting the predefined inclusion criteria. Six records were sought for full-text retrieval and were subsequently assessed for eligibility; all six were excluded following detailed evaluation. Two studies were excluded due to an inappropriate patient population (e.g., inclusion of mixed autoimmune cohorts without SLE-specific analyses), and one study was excluded because HCQ was not evaluated as an exposure of interest. Three studies reported breast cancer-specific outcomes in women with SLE; however, these were excluded because HCQ exposure was treated as background therapy or a covariate rather than as an independent exposure, or because effect estimates specific to HCQ use could not be extracted. No additional records were identified through citation searching or other sources. Consequently, no studies met the inclusion criteria, and no studies were included in the final systematic review (Figure 1).

Characteristics of the Included Articles

Because no studies met the predefined inclusion criteria, a meta-analysis was not performed. Consequently, quantitative synthesis and formal assessment of statistical heterogeneity were not feasible. The findings are therefore presented descriptively, emphasizing the absence of eligible studies. To enhance transparency, the characteristics of the six studies assessed at the full-text stage and subsequently excluded are summarized in Table 1.

**Table 1:** Studies Excluded After Full-Text Review and Reasons for Exclusion Characteristics of studies assessed at the full-text stage and excluded from the systematic review, with explicit reasons for exclusion, are summarized below. These studies were excluded because they did not meet the predefined eligibility criteria, including the absence of HCQ as an independent exposure, the absence of breast cancer-specific outcomes, or the inclusion of heterogeneous patient populations. The table is provided to enhance transparency and does not imply eligibility for inclusion or quantitative synthesis. SLE, Systemic lupus erythematosus; CLE, Cutaneous lupus erythematosus; HCQ, Hydroxychloroquine; SIR, Standardized incidence ratio

First author (Year)	Country / Data source	Study design	Population	Cancer outcome(s) reported	HCQ exposure handling	Reason for exclusion
Sultan et al. (2000) [14]	United Kingdom (single-center cohort)	Prospective cohort	SLE patients (n = 276)	Overall malignancy and selected site-specific cancers (breast cancer cases reported)	HCQ reported descriptively only	HCQ not treated as independent exposure; no effect estimates
Tallbacka et al. (2018) [15]	Finland (Finnish Cancer Registry)	Long-term cohort (>25 years)	SLE patients (n = 205)	Overall and site-specific cancers (breast cancer SIR reported)	HCQ not analyzed	No medication-specific analysis; HCQ not examined as exposure
Guo et al. (2020) [16]	China (hospital cohort)	Nested case-control	SLE patients (n = 5,858)	Overall cancer risk (breast cancer not isolated)	HCQ analyzed vs non-HCQ	Cancer outcome not breast-specific; exposure-outcome not site-specific
Westermann et al. (2021) [17]	Denmark (nationwide registries)	Population-based cohort	CLE and SLE patients (n = 5,310)	Overall and site-specific cancers (including breast cancer)	HCQ not assessed	Did not evaluate HCQ exposure; cancer risk attributed to disease status only
Zhang et al. (2022) [5]	Multi-country cohorts	Systematic review and meta-analysis	SLE patients (~247,000)	Overall and multiple site-specific cancers	No medication-level analysis	Review-level synthesis; no primary HCQ exposure data
Ichinose et al. (2024) [18]	Japan (LUNA registry)	Historical cohort	SLE patients (n = 704)	Overall cancer incidence (gynecologic cancers predominant)	HCQ treated as covariate	Primary exposure was calcineurin inhibitors; HCQ not evaluated independently

Quality Assessment Results

A formal quality assessment was planned using established risk-of-bias tools for observational studies. However, as no studies were included in the review, quality assessment was not performed.

Discussion

In this systematic review, we sought to evaluate the association between HCQ use and breast cancer risk in women with SLE. Despite an extensive and systematic search of multiple databases and the application of clearly predefined eligibility criteria, no studies directly addressed this research question. This finding highlights a critical evidence gap in the current literature.

Previous epidemiological studies have consistently examined cancer risk in patients with SLE, demonstrating an increased risk of overall malignancy and hematological cancers, while suggesting a reduced or unchanged incidence of hormone-sensitive cancers such as breast cancer compared with the general population [17,19,20]. However, these studies have largely focused on SLE as the primary exposure and have not evaluated the potential modifying effect of specific long-term treatments. In particular, HCQ - despite being a cornerstone therapy for SLE - has typically been treated as background medication or as a covariate rather than as an independent exposure of interest [21].

The absence of eligible studies is notable given the widespread and prolonged use of HCQ in women with SLE. HCQ is often prescribed from early in the disease course and continued for decades, resulting in substantial cumulative exposure [22,23]. Moreover, experimental and oncological research has suggested that HCQ may influence cancer biology through mechanisms such as autophagy inhibition, immune modulation, and effects on the tumor microenvironment [24,25]. These biological plausibility signals further underscore the importance of evaluating the long-term cancer-related safety profile of HCQ in real-world clinical settings.

Several structural factors may explain the lack of direct evidence identified in this review. First, HCQ is widely regarded as standard-of-care therapy in SLE, making it challenging to define appropriate unexposed comparator groups in observational studies [18,26,27]. Second, breast cancer remains a relatively infrequent outcome in SLE populations, limiting statistical power for drug-specific analyses [28]. Third, pharmacoepidemiological studies in SLE have historically prioritized the evaluation of cytotoxic immunosuppressive agents with established carcinogenic potential, such as cyclophosphamide, rather than immunomodulatory agents perceived as safer [29].

Importantly, the absence of evidence identified in this review should not be interpreted as evidence of no association between HCQ use and breast cancer risk. Instead, it reflects a gap in study design and analytical focus within the existing literature. Well-designed longitudinal cohort studies and nested case-control analyses that incorporate time-varying HCQ exposure, cumulative dose, and latency considerations are needed to address this clinically relevant question. Linking rheumatology registries to population-based cancer registries may offer a feasible approach to overcoming current methodological barriers.

Although no eligible studies were identified, this systematic review highlights a specific and previously unarticulated gap in the epidemiological literature: the absence of studies that evaluate HCQ as an independent exposure in relation to site-specific cancer outcomes, particularly breast cancer, in women with SLE. Existing malignancy studies in SLE have primarily focused on disease-related cancer risk or the effects of cytotoxic immunosuppressive therapies, with HCQ typically treated as background therapy or a covariate. By explicitly delineating this analytic omission and the methodological constraints that contribute to it, our review clarifies why clinically relevant questions regarding long-term HCQ safety remain unanswered and identifies specific design features - such as time-varying exposure assessment and linkage with cancer registries - required for future pharmacoepidemiological studies.

This review has several limitations to consider. First, as no eligible studies were identified, quantitative synthesis and formal risk-of-bias assessment were not possible. However, this limitation reflects the current state of the evidence rather than a shortcoming of the review methodology regarding varieties of SLE outcomes and demographics [30,31]. Second, although a comprehensive search strategy was employed across multiple databases without language restrictions, unpublished data or ongoing studies addressing this question may exist. Third, the strict inclusion criteria - particularly the requirement for HCQ exposure to be evaluated as an independent variable and for breast cancer-specific effect estimates to be reported - may have contributed to the exclusion of studies that examined cancer risk more broadly. Nevertheless, these criteria were defined a priori and were necessary to address the specific research question.

## Conclusions

This systematic review demonstrates that, despite the widespread and prolonged use of HCQ in women with SLE, to our knowledge, no epidemiological studies have directly evaluated hydroxychloroquine as an independent exposure in relation to breast cancer incidence. Rather than indicating a lack of clinical relevance, this finding reflects a consistent analytical omission within the existing literature, in which HCQ is typically treated as background therapy rather than a drug-specific exposure of interest. These results delineate a clear priority for future research: the need for well-designed pharmacoepidemiological studies that incorporate time-varying HCQ exposure, cumulative dose, and appropriate comparator groups, ideally through linkage of rheumatology registries and cancer databases. Addressing this gap is essential to inform long-term safety assessment, patient counseling, and evidence-based shared decision-making in women with SLE.

## Appendices

Appendix 1: Full electronic search strategy

A comprehensive literature search was conducted to identify studies evaluating the association between hydroxychloroquine use and breast cancer risk in women with systemic lupus erythematosus. The following electronic databases were searched from inception to November 30, 2025, without language restrictions: PubMed (MEDLINE), Embase, and Web of Science Core Collection. The search strategy combined controlled vocabulary terms and free-text keywords related to systemic lupus erythematosus, hydroxychloroquine, and breast cancer.

PubMed (MEDLINE)

("Systemic Lupus Erythematosus"[Mesh] OR lupus[tiab] OR SLE[tiab]) AND ("Hydroxychloroquine"[Mesh] OR hydroxychloroquine[tiab] OR "Antimalarials"[Mesh] OR antimalarial*[tiab]) AND ("Breast Neoplasms"[Mesh] OR breast cancer[tiab] OR breast neoplasm*[tiab])

Embase

('systemic lupus erythematosus'/exp OR lupus:ti,ab OR SLE:ti,ab) AND ('hydroxychloroquine'/exp OR 'antimalarial agent'/exp OR antimalarial*:ti,ab) AND ('breast cancer'/exp OR 'breast neoplasm'/exp)

Web of Science Core Collection

TS=(systemic lupus erythematosus OR SLE OR lupus) AND TS=(hydroxychloroquine OR antimalarial*) AND TS=(breast cancer OR breast neoplasm* OR malignancy)

Additional Search Methods

Reference lists of relevant review articles and all full-text articles assessed for eligibility were manually screened to identify additional studies. No further eligible studies were identified through citation searching or other supplementary search methods.
